# Mechanical properties of low calcium alkali activated binder system under ambient curing conditions

**DOI:** 10.1038/s41598-024-63808-z

**Published:** 2024-06-06

**Authors:** Martynas Statkauskas, Danutė Vaičiukynienė, Audrius Grinys

**Affiliations:** Faculty of Civil Engineering and Architecture, Kaunas University of Technology, Studentų g. 48, 44249 Kaunas, Lithuania

**Keywords:** Alkali-activated materials, Waste ceramic brick, Waste metakaolin, Curing regimes, Ambient temperature, Civil engineering, Mechanical properties, Ceramics

## Abstract

These days, the construction industry is facing sustainability issues, leading to the selection of greener, low-carbon, alkali-activated materials. This study examines a low calcium alkali activated system composed of three constituents (ceramic brick, metakaolin waste, and phosphogypsum). The AAB compositions consist of the primary precursor, waste ceramic brick, which is increasingly (20–100 wt%) replaced with waste metakaolin. The alkaline solution was made of sodium hydroxide and water; dosage depended on the Na_2_O/Al_2_O_3_ ratio (1.00–1.36). The AAB specimens were inspected by using XRD (X-ray diffraction) and FT-IR (Fourier transform infrared spectroscopy) methods for the evaluation of mineral composition, accompanied by SEM–EDS (scanning electron microscopy & energy dispersive X-ray spectroscopy) for the analysis of the microstructure. The compressive strength after 7, 28 and 90 days, along with water absorption and softening coefficient were determined. Also, mixture calorimetry was established. The results have shown that the initial materials are suitable for producing medium-strength alkali-activated binder under ambient temperature. The maximum compressive strength was reached by using the combination of 80% CBW and 20% MKW (13.9 and 21.2 MPa after 28 and 90 days respectively). The compressive strength development was linked with the formation N–A–S–H gel and faujasite type zeolite. A higher level of geopolymerization in composition with metakaolin waste led to lower compressive strength. Consequently, binding materials with low demand of high final and especially early compressive strength could be produced under ambient temperature curing, making them more sustainable.

## Introduction

The contemporary world is rapidly developing, leading to intense urbanization. This rise in urban areas necessitates an increase in urban infrastructure, which heavily relies on concrete, the most widely consumed building material globally. However, he increased demand for concrete has a harmful impact on the environment due to production of the essential component—ordinary Portland cement (OPC). OPC is the key component of concrete and has a substantial impact on greenhouse gas emissions (cement industry generates approximately 7% of the global CO_2_ emission)^1,2^. Carbon dioxide from the production of cement comes from calcination of limestone (around 50%) at 1400–1500 °C, burning fossil fuels to generate thermal energy (around 40%) and transportation or electricity usage (around 10%)^3^. China, India, the US, and the EU countries were the most significant CO_2_ releasing countries attributed to cement industry from 2006 to 2021^4^. The harmful impact of OPC is already widely debated, however, the concrete industry is correlated with other issues. Persistent extraction of non-renewable resources (limestone, sand etc.) from quarries leads to resource depletion and decrease in the accessibility of raw materials, also harm to the landscape^5^. One way to enhance the sustainability of the construction industry is to substitute ordinary cementitious materials with alternative cement-free ones. Alkali-activated materials (AAMs) have been established to reduce CO_2_ footprint up to 50–80%, when compared to ordinary Portland cement concrete^6^, this way becoming a suitable option to replace ordinary cementitious materials. Likewise, AAMs are considered as a credible alternative to ordinary cementitious materials, projected to contribute to a greener environment, by altering by-products and wastes into valuable new building materials without additional non-renewable resources^7^.

Alkali-activated materials occasionally also labeled as ‘geopolymers’, have been broadly discussed and promoted by countless researchers around the globe over the past decade, and are reflected as a forthcoming toolkit in the development of a sustainable construction materials industry^8^. Alkali-activated material systems are formed throughout a reaction process of an aluminosilicate precursor, frequently used in powder form as by-product, residue, waste, with an alkaline solution^9^. Alkaline hydroxide, sulfate, silicate, or carbonate is one of the possible concentrated aqueous solutions to produce the alkaline activator^10^. Alkali-activated materials have a promising future due to the superior properties compared to clinker-based materials, namely higher strength, resistance to acids and sulfate and better resistance to elevated temperatures^11–13^. Although AAMs are unlikely to entirely supersede clinker-based materials, they may be an alternative composite material depending on the availability of initial materials (wastes & by-products)^14^. However, alkali-activated material systems are not ‘perfect’ and have some limitations, which must be tackled to successfully contest against clinker-based (OPC) materials. Numerous authors^15–18^ highlight main challenges including the short setting time, high drying shrinkage, efflorescence, unknown long-term durability and performance, not environmentally friendly alkali activator and elevated curing temperature; also, authors draw attention to the lack of universal specification and standard for AAMs. Nevertheless, the main benefit of alkali-activated material systems is separation from the carbonate precursors. This way the latter materials are identified as clinker-free, CO_2_ saving, environmentally friendly materials with an excellent possibility for waste utilization^19^.

Alkali-activated materials have a promising future, but there are some key aspects when it comes to producing AAMs. Primary materials (precursors) used in AAM systems are typically found in nature (natural pozzolans etc.) or in an industrial field as by-products/wastes (fly ash, bottom ash, metallurgical slags, ceramic & glass waste, rice husk ash etc.)^20^. Meanwhile, a proper alkaline solution is usually made of sodium/potassium hydroxides, silicic salts (silicates), non-silicic salts (carbonates) and water^21^. The production of an alkali-activated material system requires constituent materials, which chemical composition must contain active forms of silicon dioxide (SiO_2_) and aluminum oxide (Al_2_O_3_) or calcium oxide (CaO). In general, alkali activated material systems are classified into two main categories according to the chemical composition, more precisely calcium content (low-calcium & high-calcium)^22,23^. The hydration products of alkali-activated materials, which precursor lacks Al_2_O_3_ and SiO_2_, but is rich in CaO are tobermorite-like ‘calcium alumino silicate hydrate’ (C–A–S–H) gel. The hardened phase of this hydration product is comparable to ordinary clinker-based ‘calcium silicate hydrate’ (C–S–H). Nevertheless, if constituent materials are rich in Al_2_O_3_ and SiO_2_, three dimensional tetrahedrally interlaced ‘sodium alumino silicate hydrate’ (N–A–S–H) gels construct, which minerology is comparable to zeolites. Also, there is a probability to develop crosslinked C–A–S–H and N–A–S–H gels when the precursor is rich in all three latter chemical compounds. According to Duxson et al.^24^ alkaline activation process is conducted throughout four stages: (1) dissolution, (2) condensation, (3) polycondensation and (4) crystallization. The first reaction process happens when amorphous aluminosilicates dissolve under a high pH condition. The second stage is precipitation of the released silicate and aluminate species into oligomers, which take place swiftly after being dissolved. The third stage is formation of alkaline aluminosilicate hydrate gel. Finally, gels transition into Si-richer phases by integrating more Si as the reaction advances, or into more systematic production by crystallization under firm reaction and curing conditions^19,25^.

The performance of alkali-activated material systems is dependent on the rate of chemical reaction, and with adequate curing regimes (temperature, humidity & time) it is easier to monitor the rate of chemical reaction. The curing conditions (regime) should therefore be chosen very carefully as it can have a dual effect on the properties of the alkali-activated material. Depending on the initial material (precursor) elemental composition, curing regimes typically take place in heat or ambient (room) temperature conditions^26^. Heat (thermal) curing usually has a huge positive impact in early AAM compressive strength since increased curing temperatures rushes the reaction kinetics geopolymerization and benefits the dissolution of the active species^27^. Although, continuous thermal curing can cause rapid moisture loss, negatively affecting other mechanical properties. Komnitsas et al.^28^ advises to choose the curing regime carefully, because exceeded curing temperature (> 150 °C) and time (> 48 h) creates a risk of shrinkage and microcracks in the conglomerate structure by rapid dihydroxylation in the AAM gel. The curing process is correlated to the properties of alkali-activated materials. Currently, there are five most used curing regimes: ambient temperature, water immersion, wrapping (sealing), thermal (oven), microwave. One of the main reasons of the ambient temperature curing regime being superior to other regimes because it produces lower CO_2_, not costing any additional energy resources; also, it resembles the curing conditions of clinker-based materials conducted on-site^29^. Ambient curing regime is described with the temperature of 20–30 °C and RH of 40–95%. The precursor chemical composition plays a significant role in ambient curing, and more commonly precursors rich in calcium are cured this way. High calcium based AAM systems share similar hydration kinetics with ordinary Portland cement systems and do not require long and high temperature thermal curing^30^. Several researchers position that the existence of OPC in the AAM composition removes the thermal curing requirement^31^. The use of water immersion curing regime is also dependent on the precursor compositional content, as the contrast among low and high calcium-based systems has been primarily linked to reaction kinetics and the development of C–S–H. Normally, water curing is described as immersion of alkali samples in hot (65–95 °C) or ambient (20–25 °C) temperature water^26,32^. According to Sajedi and Razak^33^, water immersion curing can lead to the transfer of cations from the AAM matrix into water, this way causing early leaching and noticeable loss of compressive strength. The latter phenomenon was also conducted by Provis^34^, as a low calcium-based systems are correlated with the dilution of reaction (pH reduction), leaching and strength decrease. However, water curing regime for high calcium based AAMs can initiate an improvement of durability properties (strength, lower permeability etc.)^35^. In general, wrapping (sealing) curing regime is intended to reduce the evaporation rate by covering material surface, thus establishing an envelope to prevent moisture exchange with the environment^34^. There are a couple of methods to envelope the material such as coated cast, wet burlap, but more often plastic film (polyethylene, nylon) covering^36^. Main benefits of this curing method include low-cost, possibility to cure in-situ and reduced cracking due to reduced autogenous and drying shrinkage^26,37^. Thermal curing regime is favorable for the alkali-activated systems with low amounts of calcium^34^. Throughout this curing process, the increase in temperature intensifies the reaction and dissolution of oxides and, ultimately, accelerates the kinetics of geopolymerization reaction^38^. This phenomenon is more effective in the early curing stage, thus higher early strength performances are reached. Nonetheless, thermal curing conditions must be limited, because thermal curing (> 80 °C) when curing duration exceeds 24 h period can cause negative outcome—microcracks^39^. One of the shortcomings of thermal curing is that this curing regime requires additional energy resources to create these curing conditions, this way additional CO_2_ is emitted. Conventional thermal curing is characterized by long periods of curing time and high energy consumption, but recently, an alternative curing regime with relatively short period of curing time and major energy saving has been introduced as microwave curing^40^. This curing regime does not require thermal energy to be transmitted from the exterior of the specimen to the center through a thermal gradient^26^. According to Somaratna et al.^41^, microwave energy is distributed uniformly throughout the specimen matrix by molecular level interactions with the electromagnetic field; thus, electromagnetic energy is converted to thermal energy, which in turn is used to enhance reaction kinetics and increase strength performance. The latter curing regime significantly reduces the relatively long (up to 6 h) curing time (up to 1 h); also, the power of the microwave can influence different compressive strength results^42^.

The aim of the present research is to scrutinize low-calcium alkali-activated binder (AAB) system mechanical properties under ambient curing conditions. The alkali-activated binders consist of primary precursor (waste ceramic brick) which is gradually replaced with manufacturing by-product (waste metakaolin), while a minor content of CaO is incorporated (phosphogypsum). Every stage of alkali-activated binder development under ambient curing regime is explored in the AAM system to study the interaction of initial materials, as well as relationship between physical–chemical, mechanical properties, and microstructure.

This research is a significant contribution to the recycling of ceramic brick & metakaolin waste in building materials through its activation along with phosphogypsum. In contrast to other authors’ studies^13,31,35^, this study uses production waste instead of natural resources; also, the curing of the alkali activated binder is completed at ambient temperature regime. There is a possibility that the object studied could contribute to the consumption of the latter waste materials in landfills and provide an alternative clinker-free material that requires less energy and natural resources.

## Experimental procedures

*X-ray fluorescence (XRF) analysis*. The oxides (elemental) compositions of the constituent materials were established by using X-ray fluorescence method. Laboratory equipment—an XRF spectrometer “Bruker X-ray S8 Tiger WD with a Rh tube with the energy of up to 60 eV.

*Laser diffraction*. The constituent materials parameters (density, particle size distribution from 0.1 to 500 µm and specific surface area) were inspected by using laser diffraction (dry method). Laboratory equipment—A CILAS 1090 LD laser scattering particle size analyzer.

*Scanning electron microscopy*. The microstructures of constituent materials and zeolitic creations were inspected by applying scanning electron microscopy (SEM). Laboratory equipment—a high-tenacity SEM “FEI Quanta 200 FEG” with a Schottky field emission gun (FEG). Also, the chemical composition was investigated with the Bruker Quad 5040 EDS detector (123 eV).

*Compressive strength test*. The mechanical properties of alkali-activated binders were examined by applying compressive strength performance test after 7, 28 and 90 days according to EN 12390-4:2019^43^. Cubic alkali-activated binder specimens with dimensions of 20 × 20 × 20 mm was tested. Laboratory equipment—Zwick Z100 universal analysis machine with a testing speed of 0.5 mm/min.

*X-ray diffraction (XRD) analysis*. The mineralogy of constituent materials and alkali-activated binder hydration products were assessed by using X-ray diffraction method. Laboratory equipment—“D8 Advance” diffractometer (Bruker AXS, Karlsruhe, Germany) performing at the tube voltage of 40 kV and tube current of 40 mA. The X-ray beam was sorted with a Ni 0.02 mm filter to pick the CuKα wavelength. The powder X-ray diffraction models were recognized with recommendations accessible in the PDF-2 database. The quantitative analysis of the material, carried out by the Rietveld crystal structure refinement method (TOPAS 4.1 program).

*Fourier transform infrared spectroscopy*. The mineralogy validity of AAB products of synthesis was verified by using Fourier transform infrared spectroscopy (FT-IR) method. Laboratory equipment—Perkin “Elmer FT-IR System” spectrometer. 1 mg of the matter was mingled with 200 mg of KBr and compressed in a forming press beneath vacuum for the IR investigation.

*Semi-adiabatic calorimetry test method*. Alkali-activated binder paste hydration temperatures were evaluated by using the semi-adiabatic calorimetry test procedure, based on EN 196-9:2010^44^. Laboratory equipment—8 channel USB TC-08 Data Logger and a K-type thermocouple (temperature measurement in range from − 270 °C to + 1300 °C).

*Water absorption and softening coefficient*. The water absorption and softening coefficient are crucial indexes for estimating alkali-activated binder durability and water resistance. Softening coefficient is a ratio between saturated and dry specimen compressive strength established by the following Eq. (1):

1 $$K=\frac{{C}_{w}}{{C}_{d}}$$

where ${C}_{w}$—compressive strength significance after soaking in water for 24 h; ${C}_{d}$—compressive strength value after 24 h drying.

The water absorption—the ratio of the water absorbed, and dry specimen established by the following Eq. (2):

2 $${W}_{a}=\frac{{M}_{i}-M}{M}\times 100\%$$

where ${M}_{i}$—weight of specimen after 24 h of saturating in water; $M$—weight of specimen after drying to constant mass.

## Constituent materials and mixtures preparation

**Figure 1:**
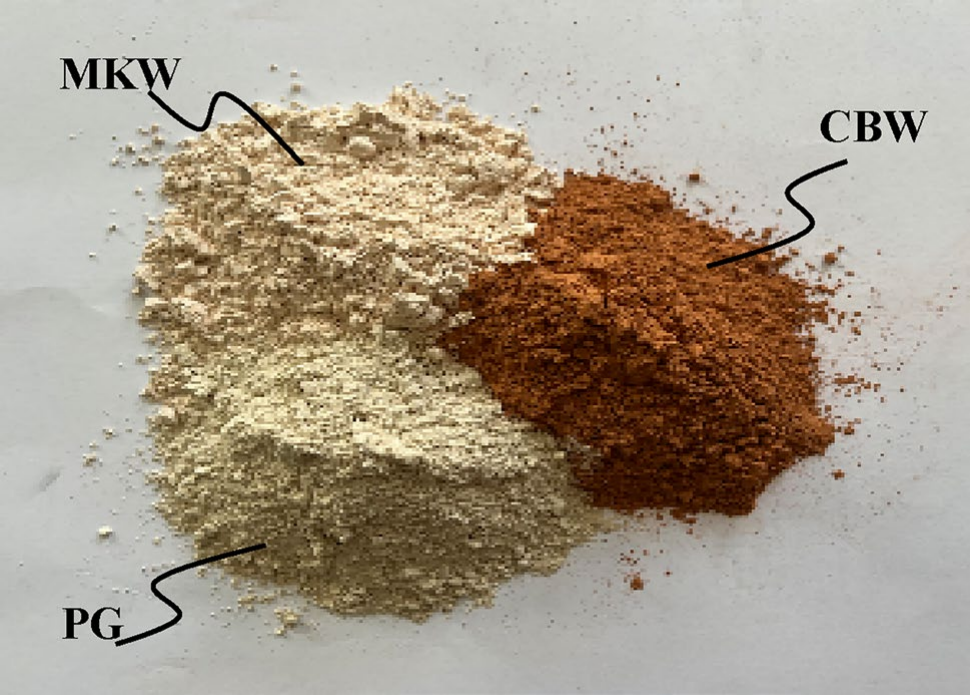
Visualization of the constituent materials: CBW—ceramic brick waste; MKW—metakaolin waste; PG—phosphogypsum.

The selected constituent materials (precursors) are rich in amorphous silicon and aluminum, and for this motive they are a perfect fit for alkali activated system. The initial materials consist of two low-calcium aluminosilicate materials (waste ceramic brick & metakaolin) and CaO source (phosphogypsum) (Fig. 1). Some materials have high pozzolanic activity with dominant crystalline phases. In this case CBW is specified with only 9.34% amorphous phase (Fig. 4b), although despite the dominant crystalline phases, the latter material has high pozzolanic activity and this could be explained by high oxide (SiO_2_, Al_2_O_3_, Fe_2_O_3_) content and the fineness of the material. Similar finding were published by Mohammed^45^. Alkaline solution consists of commercial sodium hydroxide (NaOH) pellets and a reasonable amount of liquid (water). The designated precursors are either industrial by-products or wastes, thus the secondary use of latter resources in alkali-activated binding systems is a practical option to create a sustainable building material.

**Figure 2:**
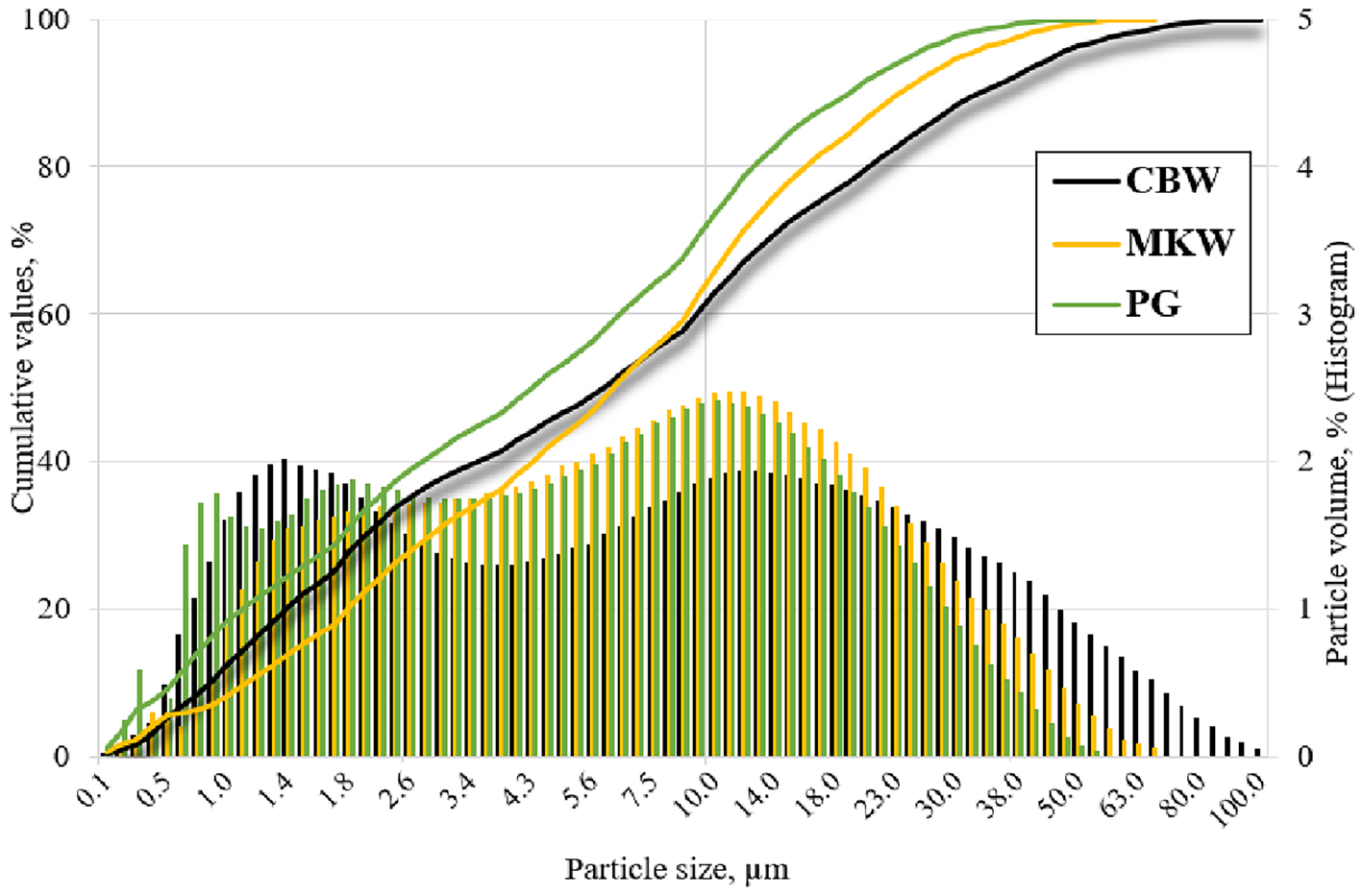
Particle size distribution of ceramic brick waste (CBW), metakaolin waste (MKW) and phosphogypsum (PG).

**Figure 3:**
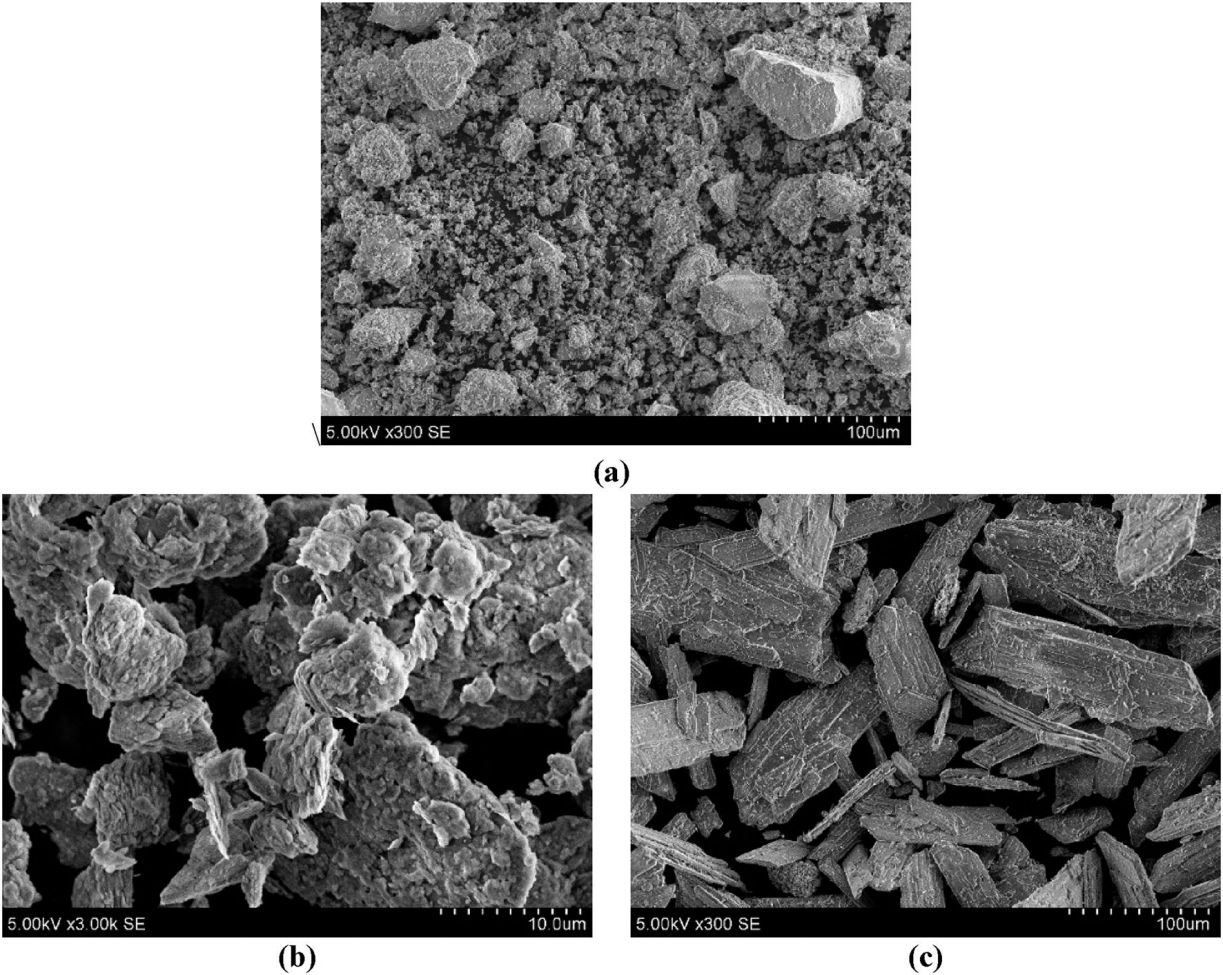
The scanning electron microscopy images of precursors: (**a**) ceramic brick waste (CBW), (**b**) metakaolin waste (MKW), (**c**) phosphogypsum (PG).

*Ceramic brick waste (CBW)*. Ceramic brick waste was found in an open dumping ground in Klaipėda, Lithuania. Ceramic brick waste of various sizes up to 15 cm was pounded with a jaw crusher to reach an average particle size of about 1.0 cm. Later, crushed ceramic brick particles were placed in a laboratory ball mill for 2 h, to increase powder fineness (specific surface area). As the years go by, the amount of ceramic brick waste, generated in landfills has been increasing all around the globe; according to 2019 data, the US and Canada spawns 170 and 17.3 million tons, respectively)^46^. Key parameters: density—2.77 g/cm^3^; specific surface area—497.96 m^2^/kg; mean particle diameter—12.89 µm; particle size distribution—in range of 0.1—130 µm (Fig. 2); the highest number of uniform particles—2.01% at 1.4 µm; particle shape—sharp-edged (Fig. 3a); main oxides (Table 1)—SiO_2_ (70.49%) and Al_2_O_3_ (13.24%).

**Table 1:** Ceramic brick waste, metakaolin waste and phosphogypsum oxides composition, wt%

Oxide	SiO_2_	Al_2_O_3_	Fe_2_O_3_	K_2_O	CaO	MgO	Na_2_O	TiO_2_	P_2_O_5_	SO_3_	Cl	Other	LOI	Total
CBW	70.49	13.24	4.72	3.72	3.67	2.13	0.88	0.69	0.15	0.10	0.03	0.18	0.85	100
MKW	49.34	32.79	0.79	0.71	0.69	0.28	14.86	0.36	0.08	0.07	–	0.03	1.84	100
PG	0.43	0.07	0.12	0.04	39.16	0.05	–	0.18	0.60	56.74	–	2.61	6.51	100

*Metakaolin waste (MKW)*. Metakaolin waste was taken from expanded glass granule company UAB “Stiklo poras”, Lithuania. Throughout the production process of expanded glass granule, kaolinite clay powder is applied to participate as a material for anti-agglomeration, this way forming an industrial by-product—waste metakaolin^47^. Waste metakaolin is polluted with expanded glass particles, for this reason it has a different oxide composition (higher amount of Na_2_O), when compared to conventional metakaolin. Key parameters: density—2.54 g/cm^3^; specific surface area—565.26 m^2^/kg; mean particle diameter—10.34 µm; particle size distribution—in range of 0.1—66 µm (Fig. 2); the highest number of uniform particles—2.47% at 12.0 µm; particle shape—plate-like stratified (Fig. 3b); main oxides (Table 1)—SiO_2_ (49.34%), Al_2_O_3_ (32.79%) and Na_2_O (14.86%).

*Phosphogypsum (PG)*. Phosphogypsum was received from a fertilizer company AB “Lifosa”, Lithuania. Essentially, phosphogypsum is an industrial by-product of orthophosphoric acid manufacture^48^. The latter by-product is produced very intensively in Lithuania; thus, it needs to be utilized. Previous studies^49^ have shown that quite small content of phosphogypsum (5%, wt. of precursor) incorporation in alkali-activated system accelerates the geopolymerization process and adds up compressive strength. Key parameters: density—2.51 g/cm^3^; specific surface area—516.32 m^2^/kg; mean particle diameter—11.01 µm; particle size distribution—in range of 0.1—53 µm (Fig. 2); the highest number of uniform particles—2.41% at 10.0 µm; particle shape—prismatic crystal with cracked ends (Fig. 3c); main oxides (Table 1)—SO_3_ (56.74%) and CaO (39.16%).

**Figure 4:**
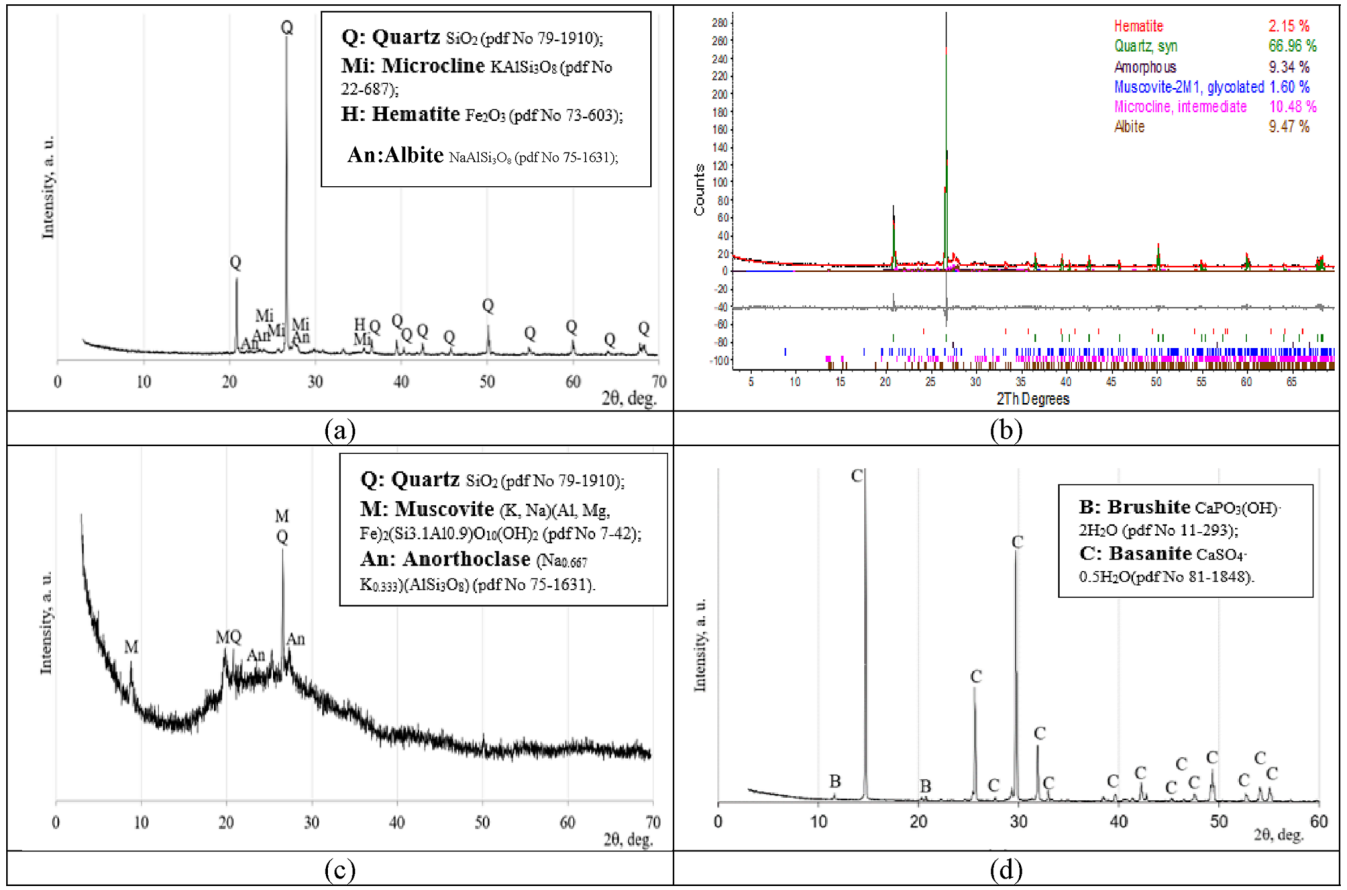
Constituent materials X consistency with Vicat apparatus ray diffraction pattern: (**a**,**b**) ceramic brick waste (CBW), (**c**) metakaolin waste (MKW), (**d**) phosphogypsum (PG).

The mineralogy of ceramic brick waste was primarily based on the presence of quartz with a small amount of microcline and hematite compounds (Fig. 4a). Metakaolin waste was mainly based on quartz, muscovite, and anorthoclase crystalline compounds (Fig. 4c). Basanite and tiny amounts of brushite were detected in the mineral composition of phosphogypsum (Fig. 4d).

**Figure 5:**
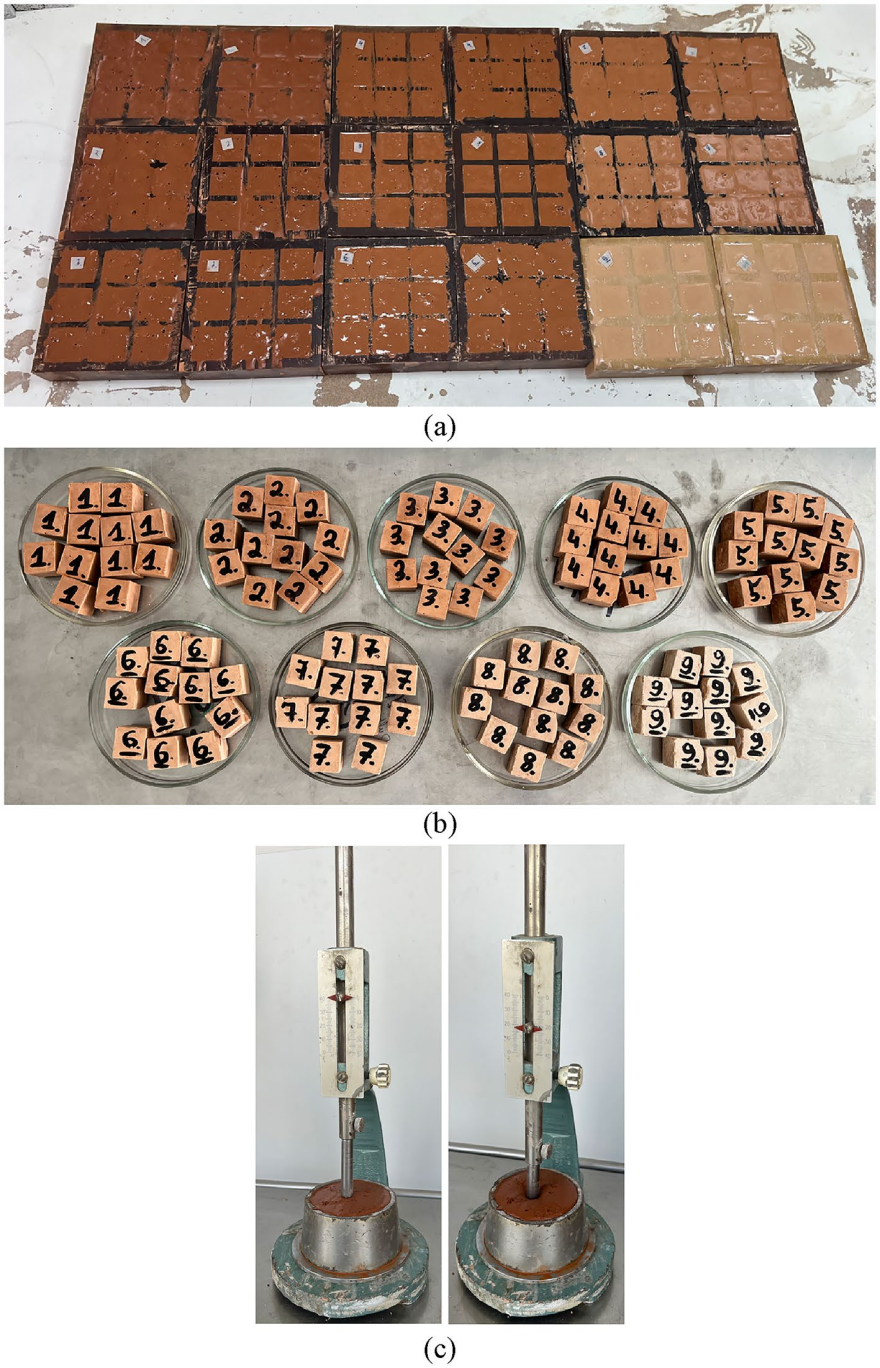
Alkali-activated binder: (**a**) freshly cast specimens; (**b**) specimens after 90 days of curing; (**c**) mixture consistency with Vicat apparatus and a plunger.

*Mixing, molding, and curing*. Alkali-activated binder mixes were prepared with stand mixer with bowl “HENSKE HM9118A-GS”. Main parameters of the mixer: capacity—3.4 L; mixing power—300 W. The preparation of AAB mixtures started by homogenously mixing dry precursors for 1 min. Later, alkaline solution was poured on the dry precursors, followed by 2 min of mixing; total mixing time—around 3 min. Cubic specimens (20 × 20 × 20 mm) were formed for the study to determine alkali-activated binder properties (Fig. 5a). Alkali-activated binder mixtures were placed in molds, then compacted on vibrating table; molds were filled in two layers, each approximately one-half of the height of the cube when compacted to avoid air bubbles in AAB matrix. To inhibit the loss of moisture, silicone molds were covered with polypropylene layer instantly after casting the mixture. After demolding, specimens were sealed with polyethylene film, to prevent further loss of moisture. Ambient curing regime was chosen for this experiment. Specimens were placed in a cabinet at ambient (room) temperature (22 ± 2 °C, 50% RH) for a total of 90 days (Fig. 5b).

A total of nine AAB system compositions were established (Table 2). The primary precursor—ceramic brick waste (CBW) was gradually changed (20, 30, 40, 50, 60, 70, 80 and 100 wt%) by waste metakaolin (MKW), however the amount of phosphogypsum (PG) stayed constant (5% wt. of CBW + MKW). As mentioned above, alkaline solution (AS) was made of NaOH granules (99% purity) and water, while maintaining the Na_2_O/Al_2_O_3_ ratio in range of 1.00–1.36; the alkaline solution and precursor (AS/Precursor) ratio was varying (0.37–0.88) in agreement to preserve identical mixture consistency in every composition. The consistency of alkali activated binders were measured by using determination of standard consistency method according to EN 196-3:2017 with Vicat apparatus and a plunger (Fig. 5c). The consistency remained the same throughout all compositions by keeping the distance between the plunger and baseplate of (20 ± 5) mm.

**Table 2:** Alkali-activated binder compositions, wt. %.

Notation	Abbreviation of composition*	AS/precursor**	w/s***	Na_2_O/Al_2_O_3_	SiO_2_/Al_2_O_3_	Raw mix SSA****	Depth of plunger penetration, mm
H1	CBW100 + MKW0 + PG5 + AS9.2/29.3	0.37	0.23	1.00	9.05	523.8	22
H2	CBW80 + MKW20 + PG5 + AS10.5/36.1	0.44	0.32	1.13	6.57	537.2	24
H3	CBW70 + MKW30 + PG5 + AS11.1/40.1	0.49	0.36	1.18	5.71	544.0	22
H4	CBW60 + MKW40 + PG5 + AS11.8/44.7	0.54	0.41	1.22	5.01	550.7	20
H5	CBW50 + MKW50 + PG5 + AS12.4/49.5	0.59	0.46	1.25	4.43	557.4	21
H6	CBW40 + MKW60 + PG5 + AS13.1/56.2	0.66	0.49	1.28	3.94	564.2	24
H7	CBW30 + MKW70 + PG5 + AS13.7/62.4	0.72	0.55	1.30	3.52	570.9	23
H8	CBW20 + MKW80 + PG5 + AS14.4/67.0	0.78	0.60	1.32	3.15	577.6	20
H9	CBW0 + MKW100 + PG5 + AS15.7/76.6	0.88	0.71	1.36	2.56	591.1	21

*Ceramic brick waste + metakaolin waste + phosphogypsum + alkaline solution: sodium hydroxide/water.

**The ratio among alkaline solution (AS) and precursor.

***w/s—water to solids ratio.

****SSA—fineness, m^2^/kg.

## Results and discussion

The low calcium alkali-activated binder was investigated in the following order according to the previously described methods. A crucial characteristic of AAB—compressive strength was established after 7, 28 and 90 days of curing at ambient regime. Besides compressive strength, a softening coefficient and water absorption were examined. Right after compressive strength test, some specimen microscopy was investigated with SEM method. Along with SEM analysis, a few specimens were milled to powder to investigate the minerology with XRD and FT-IR test methods. Also, it is mandatory to mention that fresh AAB mixture temperature change in time was investigated with semi-adiabatic calorimetry test. The resulting properties of alkali-activated binders of different compositions are defined below.

**Figure 6:**
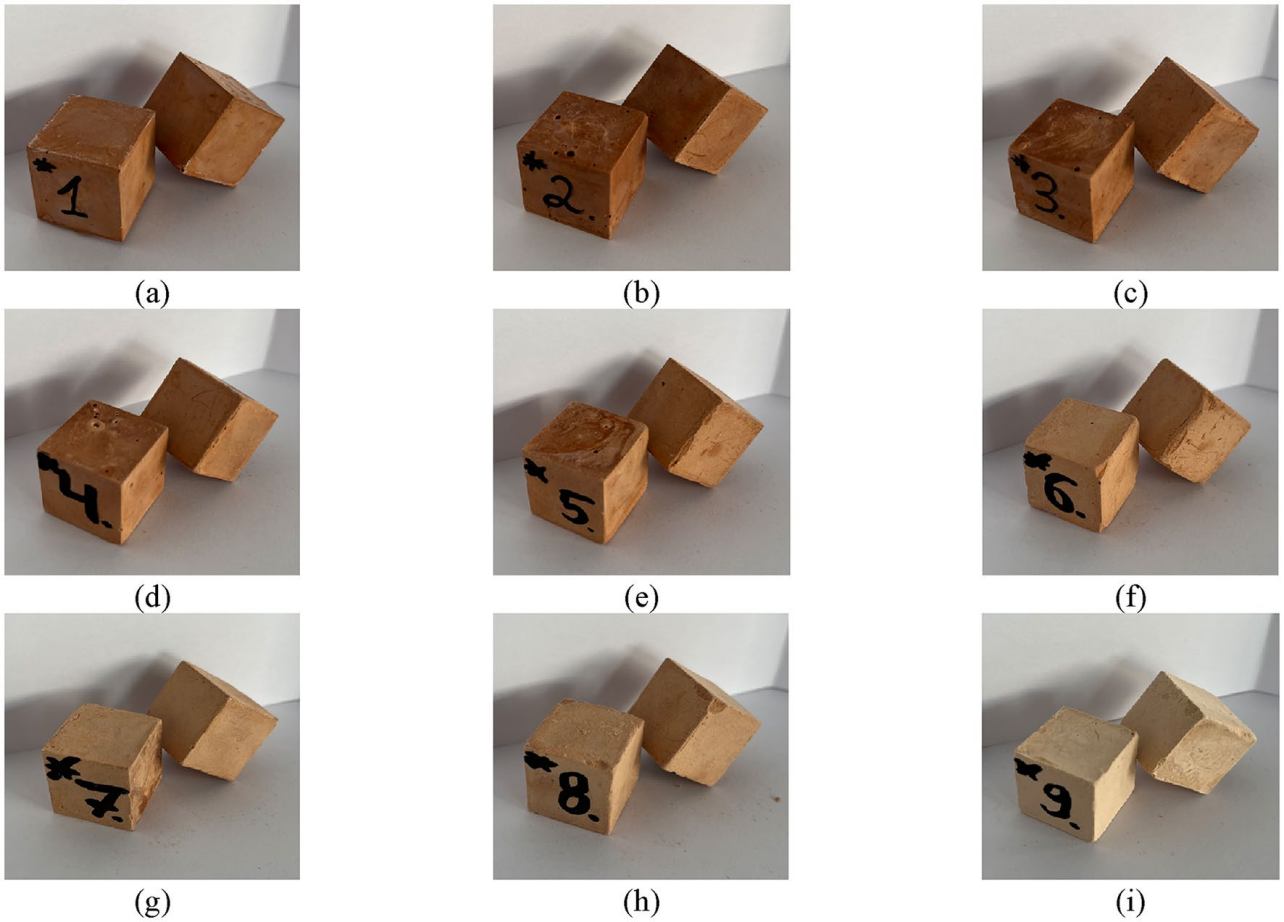
Visual examination of hardened specimens (after 90 days): (**a**) H1; (**b**) H2; (**c**) H3; (**d**) H4; (**e**) H5; (**f**) H6; (**g**) H7; (**h**) H8; (**i**) H9.

During the visual analysis of alkali-activated binder specimens (Fig. 6), no negative effects, microcracks, or efflorescence were observed, not as in our previous work^50^. The curing regimes have a huge impact on the specimen microcracking. One of the reasons visible defects occur is exceeded curing temperatures^51^. Thermal curing usually accelerates precursor dissolution with high amount of mobile alkaline leading to efflorescence and the evaporation of expelled water leading to the formation of microcracks. Thus, the assumption was made that the cracks and efflorescence phenomenon were eliminated due to low-temperature ambient curing.

**Figure 7:**
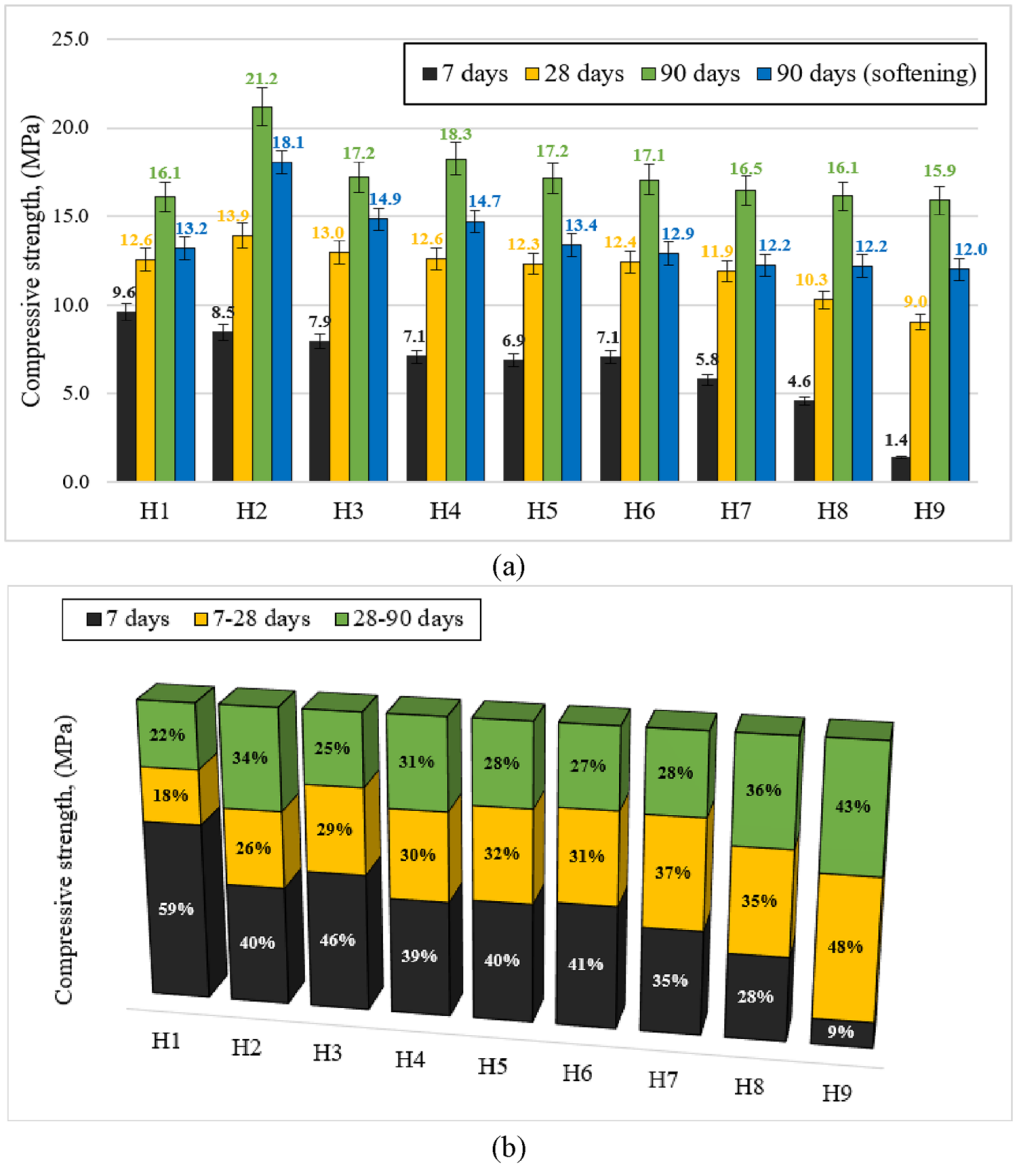
Alkali-activated binder: (**a**) the average compressive strength at different mixture proportions after 7d, 28d and 90d; (**b**) the percentage of achieved compressive strength at 7d, 7-28d and 28–90d.

*Compressive strength*. The compressive strength (CS) results at different mixture proportions and constant ambient curing regime after 7, 28 and 90 days are shown in Fig. 7a. The control specimens produced from 100% ceramic brick waste (H1) had an average CS of 9.6, 12.6 and 16.1 MPa after 7, 28, and 90 days respectively. The control composition (H1) specimens, cured under ambient temperature regime, showed an improvement on CS with the same ceramic brick waste precursor, when compared to a study conducted by Robayo et al.^27^. Although, a comparable compressive strength was obtained in research by Mahmoodi et al.^52^, where different combinations of ceramic brick waste and ceramic tiles waste were studied. The gradual substitution of ceramic brick waste with metakaolin waste in alkali-activated binder system has resulted in lower CS outcomes, due to the ambient temperature curing regime and slower geopolymerization reaction. The CS results after 7 days of curing have showed that metakaolin waste had a negative impact on early AAB strength under ambient curing conditions. Due to the increased amount of metakaolin waste, every single composition became weaker and lost its strength when compared to control composition (H1). One of the reasons for the reduction of CS after 7 days with increasing amount of metakaolin waste is the water content. To remain similar mixture consistency in all compositions, increasing amount of MKW required a higher amount of water, thus additional water content leads to increased open porosity and lower total density which leads to a decrease in early compressive strength.

Throughout the period of 7–28 curing days, every composition increased CS, but compositions which had at least slight amount of metakaolin waste, gained more than 26% of strength and the higher the metakaolin waste content, the higher the compressive strength growth during this period (Fig. 7b). The top five highest CS was achieved in compositions H2, H3, H4, H5, and H6, when compared to reference composition. The no.3–5 highest compressive strength (~ 17.2 MPa) was found in the three compositions with the combinations of 70% CBW & 30% MKW (H3), 50% CBW & 50% MKW (H5) and 40% CBW & 60% MKW (H6); in this manner, when compared to control composition the CS was about 7% higher after 90 days of curing. The no.2 highest CS (18.3 MPa) was obtained in specimens made of 60% ceramic brick waste and 40% metakaolin waste (H4); suppositionally, after 90 days of curing the CS was 13.3% higher than reference composition. The no.1 highest CS was established in the specimens with the combination of 80% CBW and 20% MKW (H2), where the AAB specimens collapsed at a maximum strength of 13.9 MPa and 21.2 MPa; in a sense, when compared to control composition 10.9% and 31.5% increase in CS was achieved after 28 and 90 days respectively. Some authors gained comparable compressive strength results by using low-calcium fly ash^53,54^ and pumice^55^. Also, some researchers achieve better compressive strength results under ambient curing regime with the incorporation of high-calcium precursors such as slag^56^, high-calcium fly ash^57^ or OPC^58^. However, it was observed that after 90 days of ambient temperature curing, the compressive strength is quite similar in every AAB composition (Fig. 7b); the CS varies from 21.2 to 15.9 MPa. One of the explanations for this phenomenon is that metakaolin waste particles (565.26 m^2^/kg) are specified with higher specific surface area (finer particles) than ceramic brick waste (497.96 m^2^/kg). Therefore, the more metakaolin waste is involved in the AAB composition, the harder it becomes to dissolve the active form silica and aluminum compounds. This is the reason why ambient temperature curing is unfavorable for AAB early strength and it needs more time to gain adequate strength^51^. Nevertheless, this phenomenon can be eliminated with the incorporation of thermal curing regime, which accelerates the reaction kinetics of geopolymerization and favors the dissolution of the active species and intensifies the formations of new compounds leading to a final compressive strength increase and especially in the early period^29^. As a matter of fact, the selected precursors show adequate results when curing at ambient temperature. Therefore, building materials that require low levels of final and early compressive strength can be cured without the need for additional energy resources, making them more environmentally friendly^30^.

**Figure 8:**
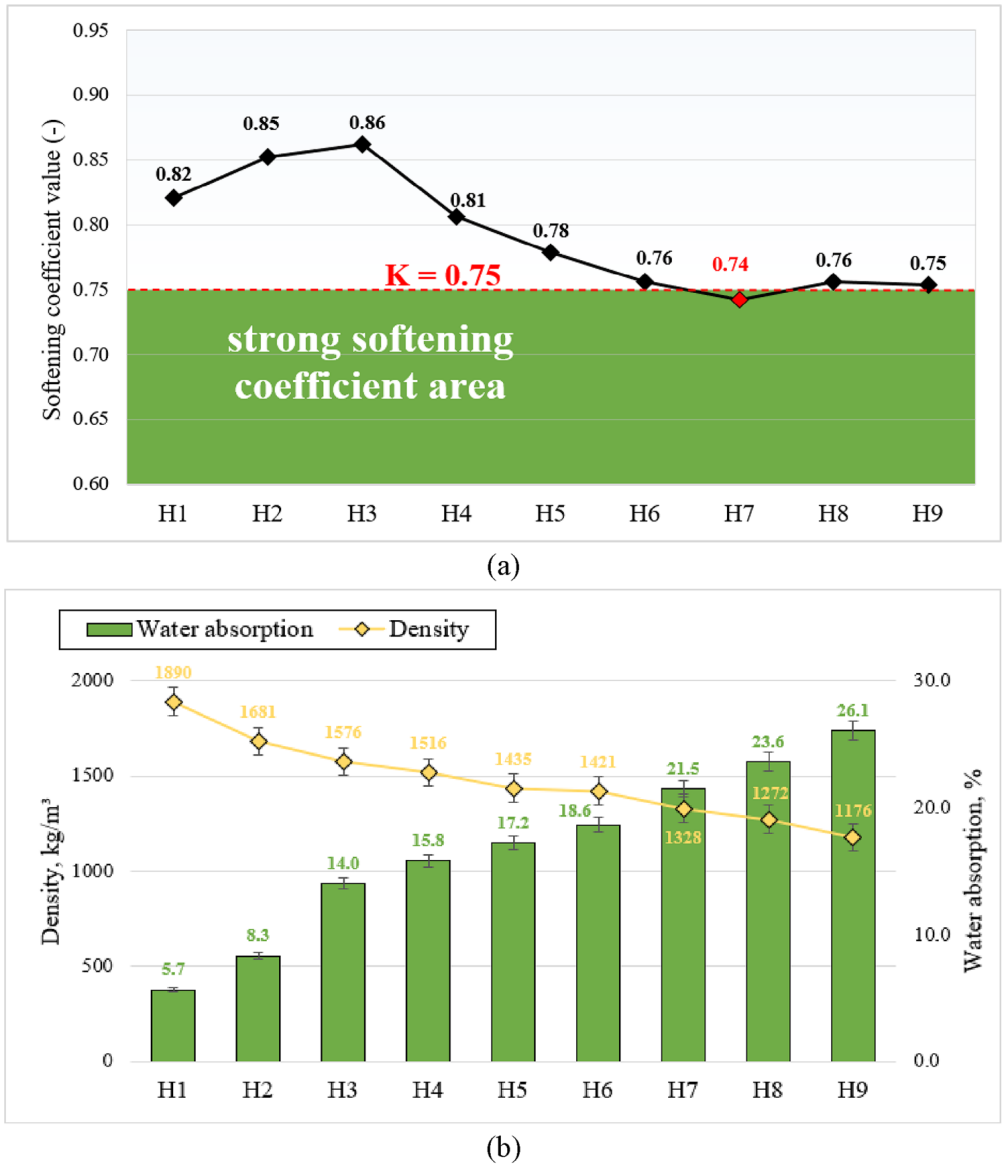
Alkali-activated binder: (**a**) softening coefficient values of alkali-activated binders after 90 days; (**b**) the relationship between specimen density and water absorption.

*Softening coefficient, water absorption and density*. To find out the effect of water on alkali-activated binder mechanical properties, specimens were exposed to water for 24 h, the softening coefficient and water absorption were used for the evaluation^59,60^. Alkali-activated binder softening coefficient values are presented in Fig. 8a, indicating the soaking manner play a side effect on the preservation of strength. The specimen is counted as weakly softened material with its softening coefficient value (K) > 0.75^61^. When evaluating softening coefficient values, it was noticed that compressive strength after 90 days links with softening coefficient. The highest softening coefficient values were obtained in two compositions (H2 = 0.85 & H3 = 0.86), where highest compressive strengths after 90 days of curing appeared. The softening coefficient increased from H1 to H3 due to the highest amount of hydration products formed, resulting in the compact microstructure, softening coefficient & highest residue compressive strength. However, the softening coefficient also depends on the precursor mineral composition and the degree of geopolymerisation of the hardened material, as demonstrated by FT-IR spectroscopy.

Also, it was noticed that with the increased amount of metakaolin waste, softening coefficient values decreased and at some point, were below strong coefficient area (H7). The relationship between AAB specimen density and water absorption is presented in Fig. 8b. Alkali-activated binder density varied from 1176 to 1890 kg/m^3^, while the specimen water absorption after water immersion varied from 5.7 to 26.1%. The decrease in specimen density is correlated with the metakaolin waste content. As mentioned before, to maintain the same mixture workability additional water content was used. Additional excess water has a crucial role in microstructural development of alkali-activated systems because it generates high open porosity, which ultimately leads to decrease in density and higher water absorption. Similar observation was made by Lizcano et al.^62^.

It was perceived that due to lower metakaolin waste bulk density and additional water content, gradual ceramic brick waste replacement with metakaolin waste ultimately leads to decrease in alkali-activated binder density and increase in water absorption content. Thus, it was observed that all above mentioned parameters correlate, the higher the metakaolin waste content in mixture compositions, the lower the material density, leading to higher water absorption, followed with lower softening coefficient value and ultimately the decrease in final compressive strength.

**Figure 9:**
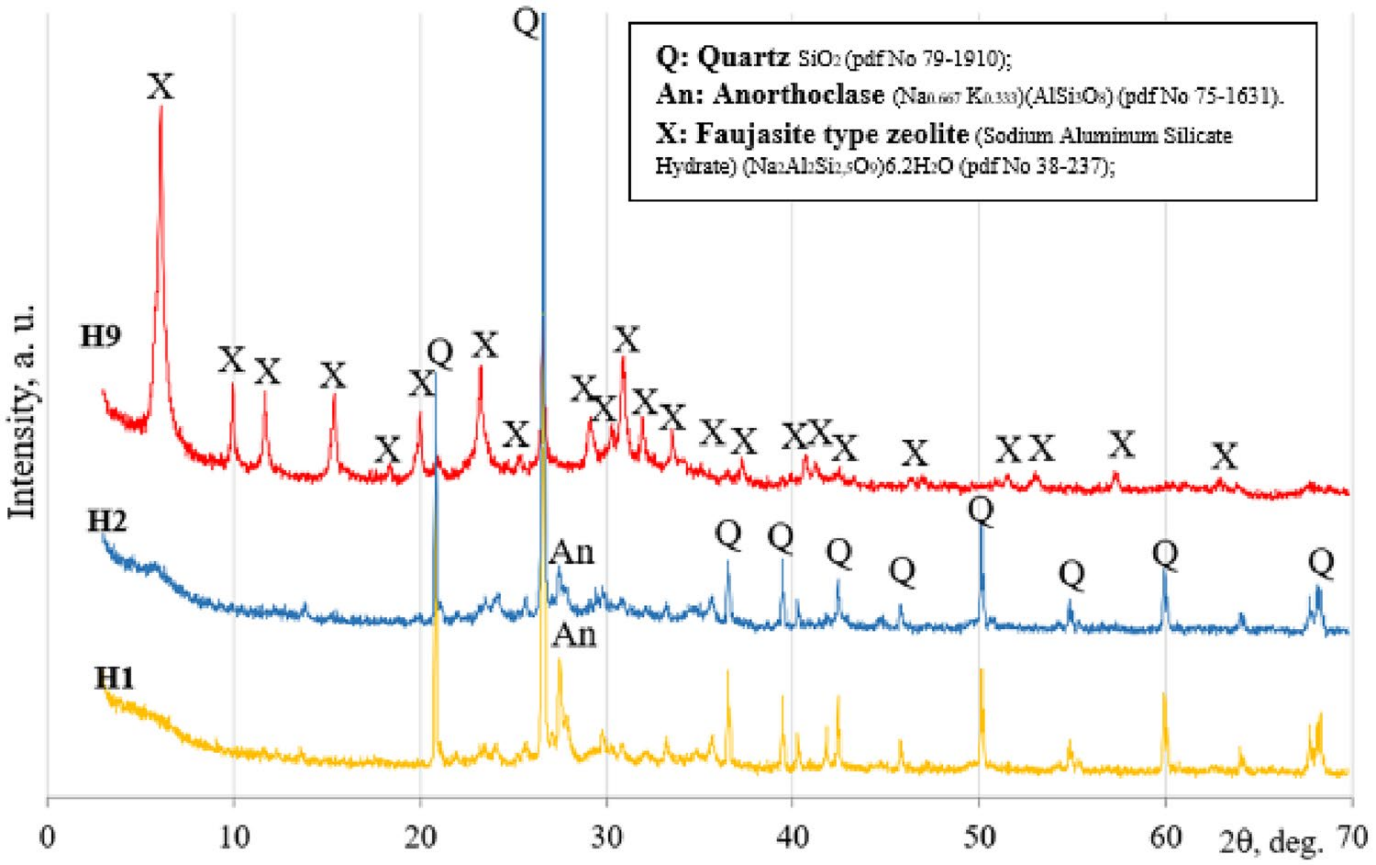
Alkali-activated binder X-ray diffraction patterns after 90 days of curing, compositions H1, H2 and H9.

*Mineral composition, according to XRD*. The minerology of alkali-activated binder specimens were assessed by using the X-ray diffraction (XRD) analysis method. The XRD patterns of three alkali-activated binder compositions are presented in Fig. 9. The XRD analysis results for the control composition composed of 100% ceramic brick waste (H1) and the composition with highest compressive strength composed of 80% ceramic brick waste and 20% metakaolin waste (H2) have shown that throughout hydration reaction such crystalline compounds as quartz (Q) and anorthoclase (An) stay unreacted^52^. Correspondingly, the XRD analysis of composition with lowest compressive strength composed of 100% metakaolin waste (H9) have shown the formation of faujasite type zeolite (X)^63^.

**Figure 10:**
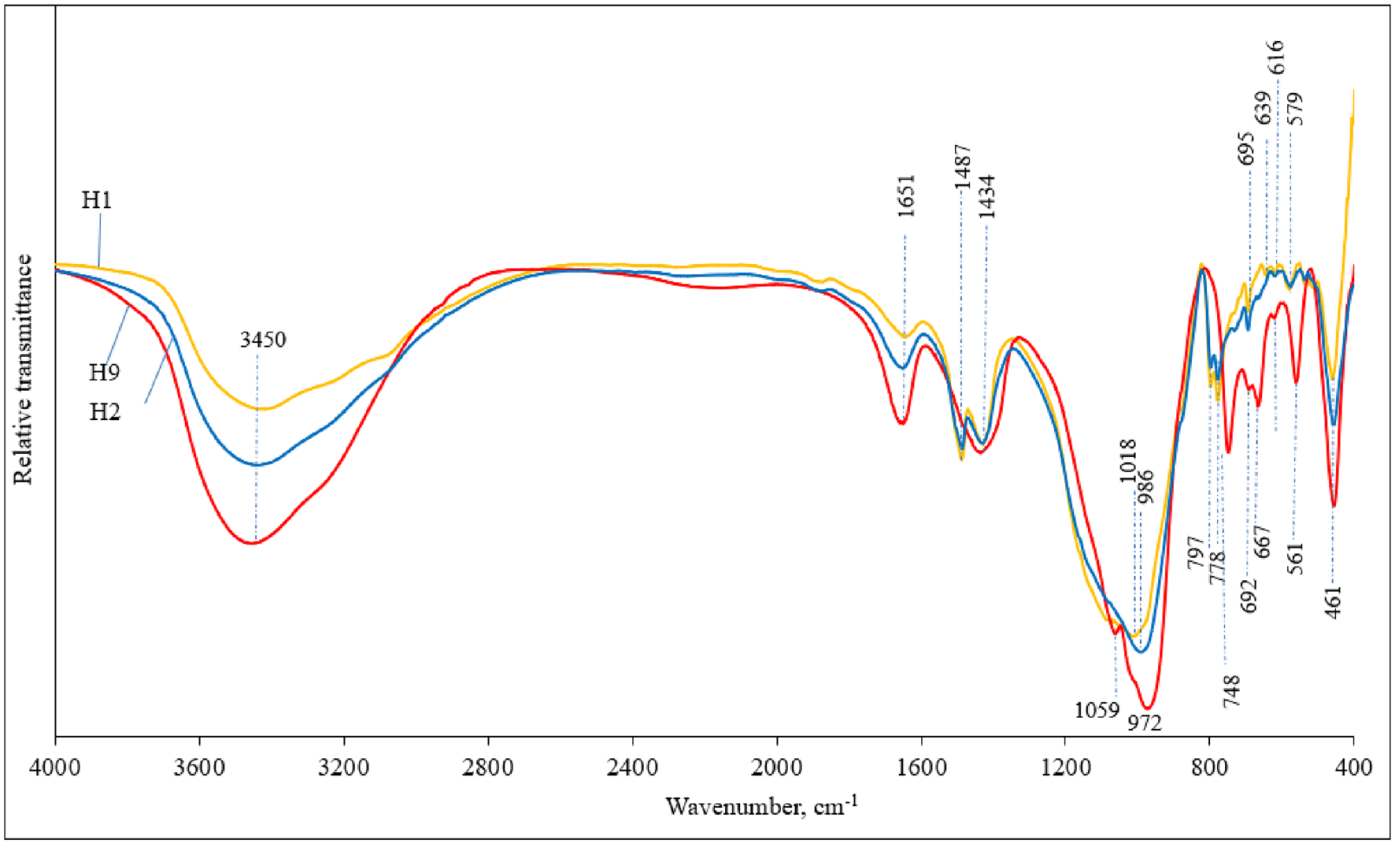
Alkali-activated binder FT-IR spectra patterns after 90 days of curing, compositions H1, H2 and H9.

*Mineral composition, according to FT-IR*. Alkali-activated binder X-ray diffraction analysis results were checked by using the Fourier transform infrared spectroscopy (FT-IR) analysis method. The FT-IR patterns of three alkali-activated binder compositions (H1, H2 & H9) are presented in Fig. 10. The Fourier transform infrared spectroscopy analysis is based on the description of functional groups. Depending on these groups, information can be acquired about the level of geopolymerization and the occurrence of diverse chemical compounds. The broad band around 3450 cm^−1^ and 1651 cm^−1^ are delegated to O–H stretching and O–H bending, correspondingly^63^. The mentioned bands intensity increased due to gradually increased metakaolin waste content in the mixtures of initial materials and higher amount of hydration water formed in alkali activation products^64^. The bands at 3450 cm^−1^, 1018 cm^−1^, 778 cm^−1^ and 797 cm^−1^ (double peak), 692–695 cm^−1^ and 461 cm^−1^ are related to the detecting of quartz^65,66^.

Moreover, bands associated with faujasite type zeolite at 3450, 1651, 1434, 972, 750, 692, 667, 561 and 461 cm^−1^ could be detected for alkali-activated binder composition (H9) made of 100% metakaolin waste^67^. Broad band at about 1018 cm^−1^ (H1) shifts toward lower wave number (986 cm^−1^ (H2) 972 cm^−1^ (H9)) and this shift is correlated to the integration of aluminum in the Si(Al)–O bond. Hence, the increasing amount of MKW in the initial mixtures led to an increase in geopolymerization level^68^. Higher levels of geopolymerization resulted in lower compressive strength values (Fig. 7). Intensified geopolymerization leads to formation of zeolitic compounds, which have porous structure with low density, as well as lower compressive strength. These findings were confirmed by Mohd Basri et al.^67^. Geopolymerization is the formation of geopolymer, during which forms an amorphous and crystalline compound. It was observed that band at about 972–1018 cm^−1^ was responsible for the main hydration product—‘sodium alumino silicate hydrate’ (N–A–S–H) gel^69^. Also, these bands overlaps with crystalline faujasite type zeolite and N–A–S–H gel. Consequently, the Fourier transform infrared spectroscopy results agree with the X-ray diffraction analysis results.

*Semi-adiabatic calorimetry*. The geopolymerization is an exothermic process containing different development stages depending on the chemical composition of precursor. The hydration process of alkali-activated binder was inspected by operating the semi-adiabatic calorimetry test method. A total of 6 distinct AAB compositions (H1, H2, H3, H4, H5 & H9) with different ceramic brick & metakaolin waste proportions were studied. An even amount of phosphogypsum (5%, wt. of precursor) was incorporated into all compositions to slightly accelerate the geopolymerization development^49^.

**Figure 11:**
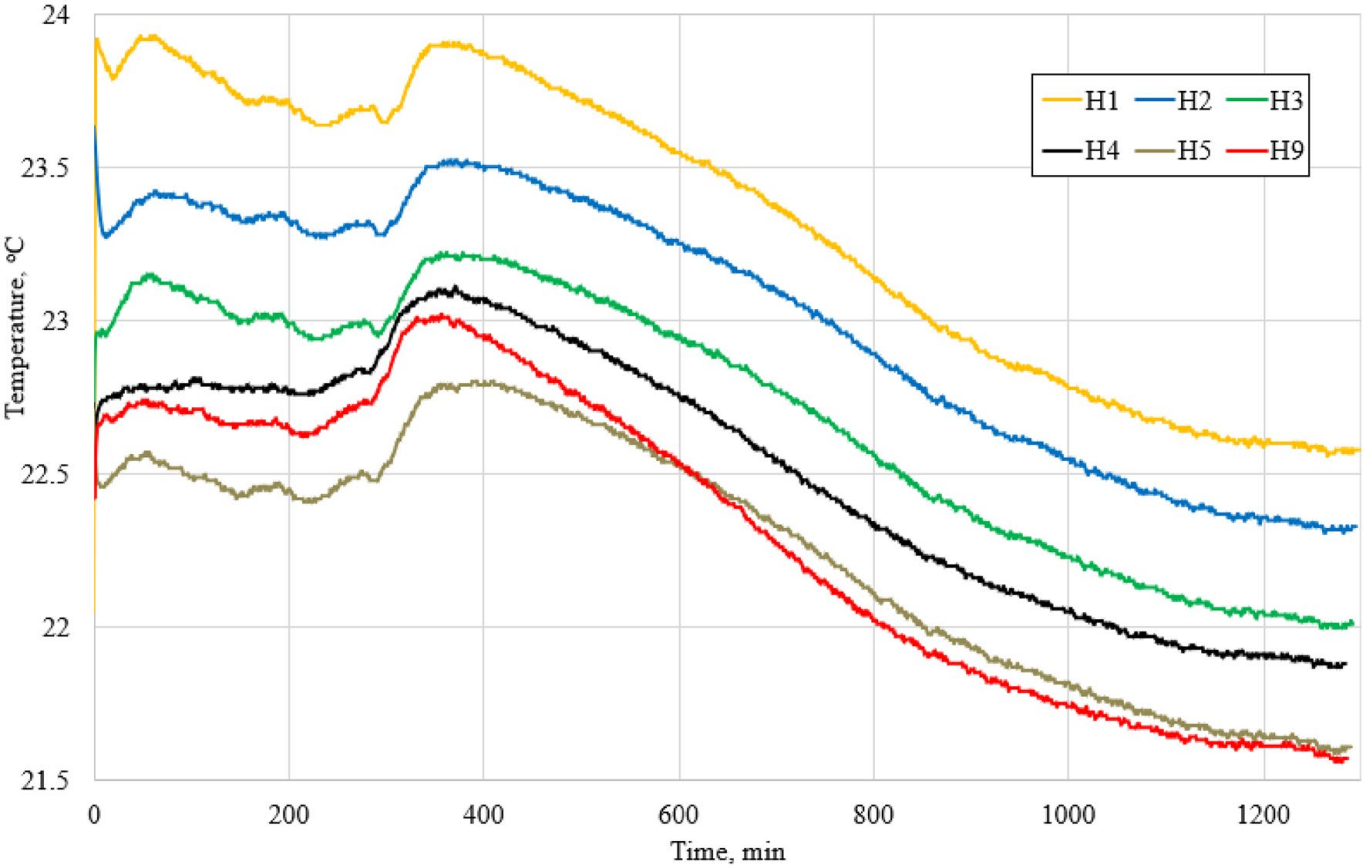
Semi-adiabatic calorimetry test results. The change in hydration process temperature of alkali-activated mix over time.

The semi-adiabatic calorimetry curves are presented in Fig. 11. The calorimetry curves have shown a similar two-stage exothermic process, mentioned in other studies^70,71^. The first peak occurred within the first 30 min after the precursors were in contact with an alkaline solution; this peak is responsible for the dissolution of the ceramic brick & metakaolin waste into silicate and aluminate monomers. The second peak is responsible for the geopolymerization process and occurred at around 330–400 min; at this point, the aluminate and silicate monomers happening to polymerize into alumino-silicate oligomers and geopolymer fragments, leading to the appearance of 3D alumino-silicate system (Si–O–Al). Generally, the latter peak is reliable for the strength of the alkali-activated material.

It is clearly noticeable that every composition have similar peaks at the same duration. Although, composition with 100% ceramic bricks waste (H1) exhibits higher amount of heat (max—23.91 °C) than the ones containing metakaolin waste. This phenomenon might be explained by the differences in Na_2_O/Al_2_O_3_ ratio. The compositions with gradually increasing amount of metakaolin waste have an increasing Na_2_O/Al_2_O_3_ ratio (H1—1.00, H2—1.13, H3—1.18, H4—1.22, H5—1.25, H9—1.36). Alkali concentration (sodium and calcium compounds) affects the degree and rate of geopolymerization. Compositions with a higher amount of ceramic brick waste (CBW) have a higher reaction rate. It has been confirmed that calcium significantly accelerates the setting of alkali activated CBW and MKW. According to Chen et al.^72^, the faster setting is associated with the promotion of geopolymer gel formation by calcium, and sodium leads to less inhibition of dissolution precursors.

**Figure 12:**
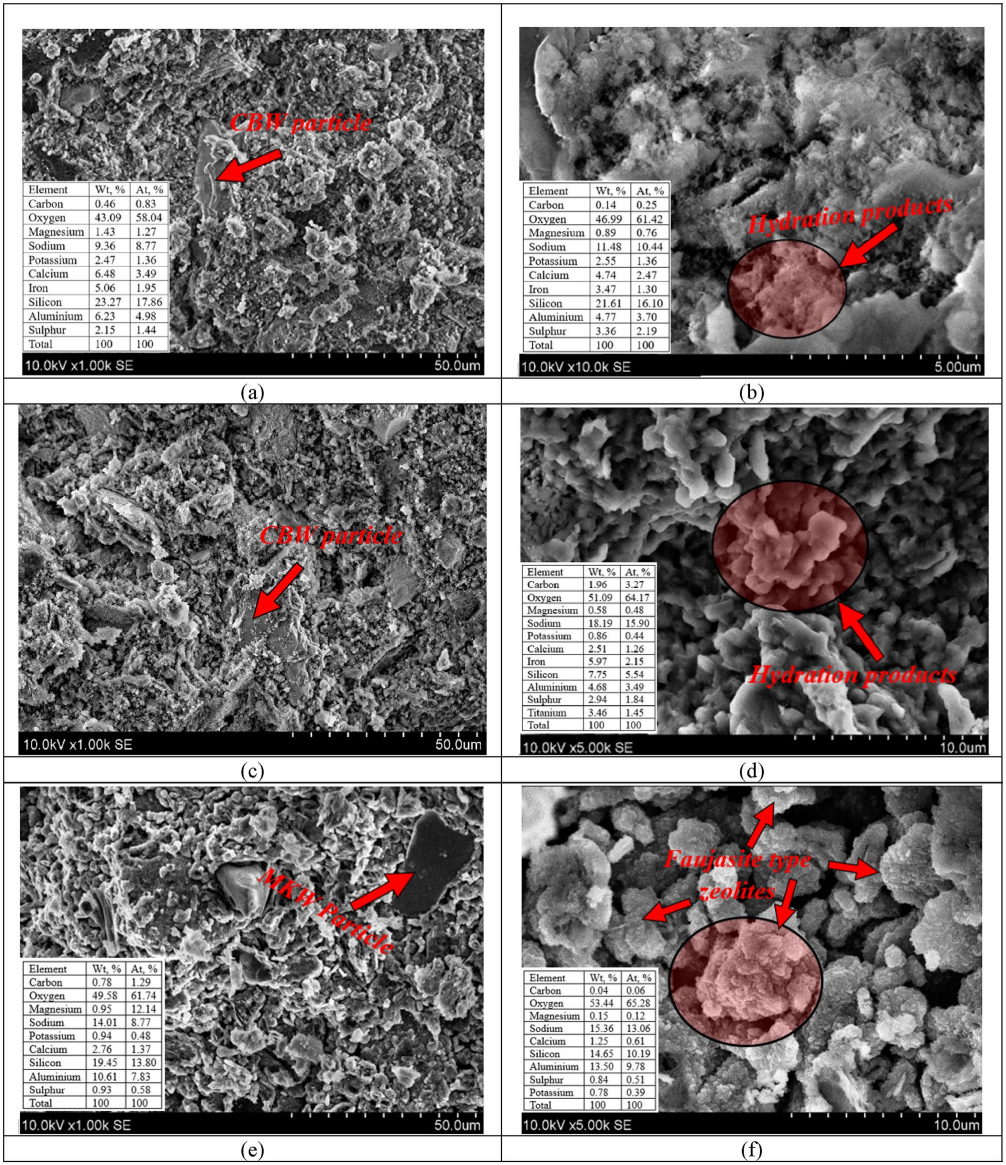
Alkali-activated binder microstructure after 90 days of curing at altered enlargement (scales: 5 μm, 10 μm, 50 μm) with EDS chemical analysis. Notes: (**a**), (**b**) H1; (**c**), (**d**) H2; (**e**), (**f**) H9.

*Microstructure, according to SEM*. The microstructure of alkali-activated binder specimens were assessed by using the scanning electron microscopy (SEM). Also, EDS (energy dispersive X-ray spectroscopy) was performed to confirm the microstructure findings. The SEM images at altered enlargement levels of three alkali-activated binder compositions are presented in Fig. 12. The hydration products (N–A–S–H gel) of AABs composed of 100% CBW (H1) and a combination of 80% CBW & 20% MKW (H2) are plainly evident^73^, as well as the unreacted ceramic brick waste particles (Fig. 12a–d). The MKW particles (Fig. 12e) had a different shape than Fig. 3b; this difference in microstructure can be explained by the origin of this material. This material is a waste (MKW) and may have a different microstructure. According to the dominant chemical elements: silicon (13.80 At. %), aluminum (7.83 At. %), sodium (8.77 At. %) and calcium (1.37 At. %), it can be said to be an MKW particle. The highest compressive strength was obtained in composition H2; this might have happened due to highly dense microstructure (Fig. 12c,d) whereas similar SEM images were obtained by Zhao et al.^74^. Moreover, the composition which lacks ceramic brick waste (H9) have formed the earlier mentioned faujasite type zeolites (Fig. 12e,f)^75^. The existence of zeolite is confirmed by EDS. In the case a significant amount of silicon (10.19 At. %), aluminum (9.78 At. %), and sodium (13.06 At. %) existed.

## Conclusions

In this study, a low calcium AAB system made from ceramic brick waste & metakaolin waste was investigated under ambient temperature curing regime. The interaction of the constituent materials was studied as well as the correlation between microstructure, physicochemical and mechanical properties.

An observation was made that it is hard to gain early-age (7 days) compressive strength under ambient temperature curing regime, when metakaolin waste is incorporated into alkali-activated binder composition. This phenomenon is due to the intensive dissolution of aluminosilicate compounds, which leads to faster geopolymerization reactions and the formation of higher crystallinity zeolites; in this case, faster geopolymerization forms a porous AAB structure with low density and lower compressive strength.

It was observed that a high content of metakaolin waste (100%) in AAB results in the formation of faujasite type zeolite. This leads to low material density, high water absorption, and a relatively low softening coefficient, ultimately resulting in low early compressive strength of the alkali-activated binder (composition H9).

The highest compressive strength (CS) was achieved in the mixture composed of 80% CBW and 20% MKW, with values of 8.5 MPa, 13.9 MPa, and 21.2 MPa after 7, 28, and 90 days of curing. The growth of CS in the latter composition was correlated with the formation of a compact microstructure composed of ‘sodium aluminosilicate hydrate’ (N–A–S–H) gel. ‘sodium alumino silicate hydrate’ (N–A–S–H) gel, which was confirmed by FT-IR analysis.

The findings of this study offer an opportunity to utilize the waste materials mentioned above, resulting in the production of clinker-free conglomerate material. This material is characterized by significant mechanical properties and low carbon emissions. The alkali-activated binder was produced using a combination of ceramic brick waste, metakaolin waste, and phosphogypsum under an ambient temperature curing regime for the first time.

Therefore, the research indicates that the selected precursors produce satisfactory results when cured at ambient temperature. This means that binding materials requiring low final and early compressive strength can be cured without additional energy resources, making them more environmentally friendly.

## Data Availability

All data generated or analyzed during this study are included in this published article.
